# Extracellular Vesicle MicroRNA in Malignant Pleural Effusion

**DOI:** 10.3390/genes13112159

**Published:** 2022-11-19

**Authors:** Samira Shojaee, Giulia Romano, Trinidad M. Sanchez, Gulmira Yermakhanova, Michela Saviana, Patricia Le, Giovanni Nigita, Federica Calore, Rachel Guthrie, Kathryn Hess, Le Kang, Theresa Swift-Scanlan, Jacob T. Graham, Najib M. Rahman, Patrick S. Nana-Sinkam, Mario Acunzo

**Affiliations:** 1 Vanderbilt University Medical Center, Department of Internal Medicine, Division of Allergy, Pulmonary and Critical Care Medicine, 1301 Medical Center Drive, Suite B187, Nashville, TN 37232, USA; 2 Virginia Commonwealth University Health System, Department of Internal Medicine, Division of Pulmonary and Critical Care Medicine, Richmond, VA 23298, USA; 3 Department of Molecular Medicine, University La Sapienza, 00161 Rome, Italy; 4 Department of Cancer Biology and Genetics, The Ohio State University, Columbus, OH 43210, USA; 5 Virginia Commonwealth University Health System, Department of Biostatistics, Richmond, VA 23298, USA; 6 Virginia Commonwealth University School of Nursing, Richmond, VA 23298, USA; 7 Oxford Respiratory Trials Unit, University of Oxford, Oxford NIHR Biomedical Research Centre, Oxford and Chinese Academy of Medical Sciences Oxford Institute, Oxford OX3 7LE, UK

**Keywords:** malignant pleural effusion, extracellular vesicle microRNA, lung cancer, breast cancer, EV-miRNA

## Abstract

Lung and breast cancer are the two most common causes of malignant pleural effusion (MPE). MPE diagnosis plays a crucial role in determining staging and therapeutic interventions in these cancers. However, our understanding of the pathogenesis and progression of MPE at the molecular level is limited. Extracellular Vesicles (EVs) and their contents, including microRNAs (miRNAs), can be isolated from all bodily fluids, including pleural fluid. This study aims to compare EV-miRNA patterns of expression in MPE caused by breast (BA-MPE) and lung (LA-MPE) adenocarcinomas compared to the control group of heart-failure-induced effusions (HF-PE). We conducted an analysis of 24 pleural fluid samples (8 LA-MPE, 8 BA-MPE, and 8 HF-PE). Using NanoString technology, we profiled miRNAs within EVs isolated from 12 cases. Bioinformatic analysis demonstrated differential expression of miR-1246 in the MPE group vs. HF-PE group and miR-150-5p and miR-1246 in the BA-MPE vs. LA-MPE group, respectively. This difference was demonstrated and validated in an independent cohort using real-time PCR (RT-PCR). miRNA-1246 demonstrated 4-fold increased expression (OR: 3.87, 95% CI: 0.43, 35) in the MPE vs. HF-PE group, resulting in an area under the curve of 0.80 (95% CI: 0.60, 0.99). The highest accuracy for differentiating MPE vs. HF-PE was seen with a combination of miRNAs compared to each miRNA alone. Consistent with prior studies, this study demonstrates dysregulation of specific EV-based miRNAs in breast and lung cancer; pleural fluid provides direct access for the analysis of these EV-miRNAs as biomarkers and potential targets and may provide insight into the underlying pathogenesis of tumor progression. These findings should be explored in large prospective studies.

## 1. Introduction

Lung and breast cancer are two of the most common cancers, with lung cancer resulting in the highest cancer mortality among men and women worldwide [1]. Diagnostic challenges related to tissue acquisition have led to increasing interest in the role of liquid biopsies in developing relevant biomarkers for the understanding of disease pathogenesis and as potential diagnostics and therapeutic targets. Pleural fluid is one potential source of liquid biopsy. Lung and breast cancer are the two most common causes of malignant pleural effusion (MPE), leading to significant morbidity and mortality. However, the sensitivity of pleural fluid cytopathological analysis in the diagnosis of MPE is limited and relies on the presence of malignant cells in the fluid and varies with tissue type [2]. For these reasons, the discovery of an extracellular biomarker in body fluids is an attractive diagnostic alternative. 

MicroRNAs (miRNAs) are endogenous non-coding RNAs of 20–22 nucleotides, regulating gene expression at the post-transcriptional level. It has been shown that select miRNAs are located in cancer-associated genomic regions, acting as oncogenes or tumor suppressors [3]. Extracellular Vesicles (EVs) are membrane-bound nano-vesicles secreted by all cell types and contain proteins, RNAs, and miRNAs, harboring biologically relevant biomarkers in the setting of MPE [3]. EVs can be found in almost all bodily fluids, including blood, urine, peritoneal, and pleural fluid [4]. EV-encapsulated miRNAs are cellular miRs that are loaded in Extracellular Vesicles (EVs) [5]. The transfer of EVs represents a novel mechanism for intercellular communication between a cell and its microenvironment. Recent studies have shown that EVs play an important role in cell-to-cell communication, inflammation, and immune modulation, especially as they relate to cancer pathogenesis, progression, metastasis, and tumor escape [6]. For example, in lung cancer, EV-encapsulated miRNAs can be used as predictive (e.g., miRNA-21, miRNA-122, and miRNA-205), diagnostic (e.g., miRNA-21, miRNA-205, and miRNA-126), and prognostic (e.g., let-7, miRNA-16, and miRNA-21) biomarkers [5]. EV-miRNAs have been studied in MPEs compared to non-malignant pleural effusions (NMPEs) in small cohorts [7,8]. These studies have shown differential expression of select miRNAs between malignant and non-malignant effusions. The number and type of dysregulated miRNAs in these studies vary significantly, with only a few miRNAs overlapping between them. These inconsistencies are likely due to heterogeneous populations with different stages of malignancy, a varying mix of malignant and non-malignant etiologies, and a lack of adjustment for additional confounding factors that could influence miRNA composition. Additionally, varying methods were used to isolate EVs and investigate miRNAs. 

This study aims to profile EV-miRNA expression patterns in MPE compared to NMPE within three homogeneous groups: MPE due to lung adenocarcinoma (LA-MPE), MPE due to breast adenocarcinoma (BA-MPE), and Heart-Failure-induced Pleural Effusion (HF-PE), which were each diagnosed based on standard clinical criteria. In this exploratory analysis, we use NanoString technology to evaluate the miRNAs within the isolated vesicles.

## 2. Materials and Methods

### 2.1. Patient Population

From a prospectively collected pleural fluid biorepository, pleural specimens from adult patients (>18 years of age) with pleural effusion referred for diagnostic and therapeutic thoracentesis were included. The study group included patients with MPE due to breast or lung adenocarcinoma proven on pleural fluid cytology or pleural biopsy. The control group included non-malignant effusions due to heart failure. HF-PE was defined as all of the following: an effusion with negative cytology, a diagnosis of heart failure, the absence of liver or renal failure, and no evidence of malignancy over a 2-year follow-up period. Patients were excluded if a concomitant second malignancy was present or suspected. Three subgroups (LA-MPE, BA-MPE, and HF-PE) were formed for analysis. The local Institutional Review Board approved this study.

### 2.2. Data and Sample Collection and Analysis

Thoracentesis was performed using a standard technique. Pleural fluids were spun at 1000× *g* for 30 min at 4 °C. Supernatants were aliquoted and stored at −80 °C. 

Patient-specific variables were obtained from chart review and stored in a de-identified secure database (REDCap).

Details of the aspiration procedure are included in Appendix A.

### 2.3. EVs Isolation

EVs were isolated from 1 mL of MPE using Total Exosome Isolation Reagent (from other body fluids) (INVITROGEN # 4484453) following the manufacturer’s protocol. EVs were thoroughly resuspended in 200 µL of PBS.

### 2.4. RNA Extraction

Thirty-five picograms of non-human miRNA spike-ins (cel-miR-248; osa-miR-414; ath-miR-159a) was mixed with each sample for RNA normalization purposes. Then, RNA was extracted with TRIzol solution (Invitrogen, Waltham, MA, USA), according to the manufacturer’s instructions, followed by an RNA extraction kit (NORGEN, Thorold, ON L2V 4Y6, Canada, #43200).

### 2.5. Nanoparticle Tracking Analysis

EVs were analyzed by nanoparticle tracking, as previously described [9].

### 2.6. NanoString nCounter Assay

Total exosomal RNA from a sub-cohort of 12 samples (4 LA-MPE, 4 BA-MPE vs. 4 HF-PE) was profiled through NanoString nCounter Human v3 miRNA Expression Assay (NanoString Technologies, Seattle, WA, USA) that allows for up to 800 different miRNA measurements in each sample concurrently. Additionally, the NanoString miRNA codeset included 6 positive and 8 negative controls to allow for the normalization of all samples based on any differences in hybridization and to set a minimum threshold miRNA count for analysis. The codeset also included 6 ligation controls to allow for the adjustment of potential ligation differences between the target miRNA and the miRtag.

For each sample, 4.0 μL of total exosomal enriched RNA was allowed to anneal with DNA “bridge oligos” that bound both the sample miRNAs and multiplexed RNA tags (miR-tags). Mature miRNAs were then joined to sequence-specific miR-tags using a Ligase enzyme and all tags in excess were removed by a subsequent enzyme clean-up step. Both capture and reporter probes were then allowed to hybridize overnight for 17 h at 65 °C with the ligated and tagged sample miRNAs as per protocol to allow the color-beaded “string” probes to bind with their sequence-specific targets. Probe excess was removed using a two-step magnetic-bead-based purification on an automated fluidic handling system (nCounter Prep Station) and target/probe complexes were immobilized on the cartridge for data collection. The nCounter Digital Analyzer collected the data by taking images of immobilized fluorescent reporter probes in the sample cartridge with a CCD camera through a microscope objective lens. For each cartridge, a high-density scan encompassing 550 fields of view was performed. Images were subsequently processed internally into a digital format (RCC files) where raw count results for each miRNA could be analyzed.

### 2.7. NanoString Data Analysis

microRNA raw data counts were analyzed with nSolver™ Analysis Software (Version 4.0; NanoString Technologies, Seattle, WA, USA). Six negative controls were used to perform background thresholding; however, it is now well known that the Negative C control is consistently inflated in many sample types and, therefore, all Negative C values were removed before calculating the negative threshold count as per NanoString recommendations. Positive controls were used to perform technical normalization to adjust any lane-by-lane variability due to differences in hybridization or binding.

After technical normalization, the data were biologically normalized by calculating the geometric mean of the top 100 miRNAs in all samples as recommended by NanoString. *p*-values were calculated using the limma package (v3.48.3) from the Bioconductor (v3.14) R (v4.1.1) project. For each pairwise analysis, we considered all significantly (*p*-value < 0.05) deregulated miRNAs and expressed at >20 counts in at least one condition (as 20 counts make up approximately the average of the expression of the negative controls in the NanoString panel) for the downstream analysis.

### 2.8. RT-qPCR Analysis

For the analysis, 3 μL of RNA was retrotranscribed by using TaqMan™ Advanced miRNA cDNA Synthesis Kit (#A28007), and Quantitative Reverse Transcription Polymerase Chain Reaction (qRT-PCR) was performed using TaqMan^®^ reagents (TaqMan™ Advanced miRNA assay # 4444964, Thermo Fisher, Waltham, Ma, USA), following the manufacturer’s instructions. All custom probes were from Applied Biosystems. The comparative Ct method for relative quantification of gene expression (Applied Biosystems, Waltham, Ma, USA) was used to determine miRNA expression levels. Samples with a Ct value of >35 were excluded from the statistical analysis. The 2^−Δct^ values were used as relative expression of the biostatistical analysis.

### 2.9. Electron Microscopy

An FEI Vitrobot (Mark IV) plunge freezer was used to prepare vitrified cryo-TEM specimens from the solution sample [10]. Cryo-TEM observation was performed on an FEI Tecnai F20 transmission electron microscope. The basic experimental setup and procedure can be found in manuscript by Gao et al. [11]. 

### 2.10. Target Prediction Analysis

All miRNA-target predictions were generated by isoTar (v1.2.1) [12]. IsoTar leverages state-of-the-art prediction tools, such as miRmap (v1.1) [13], TargetScan (v7.0) [14], PITA (v6) [15], RNAhybrid (v2.1.2) [16], and miRanda (v3.3a) [17]. IsoTar focuses solely on seed regions of 7-8 nucleotides in length (7mer-A1, 7mer-m8, 8mer), with no mismatch or G:U base pairs (wobbles). All predictions were performed using isoTar default parameters. We required a minimum consensus of four prediction tools. GO term-based functional enrichment analyses were performed based on the list of predicted targets. We retained all those GO terms that are statistically significant according to Fisher’s exact test with a *p*-value < 0.01.

### 2.11. Statistical Analysis

Simple descriptive statistics were used to describe patient demographics. Categorical variables were summarized using frequencies with percentages. Continuous variables were displayed as mean and standard deviation (SD) when normally distributed or median and interquartile range (IQR) if appropriate. Multivariable logistic regression was used to identify the statistical association of the composite of dysregulated miRNAs with MPE vs. NMPE, as compared to the association of each miRNA alone with MPE vs. NMPE. Receiver operating characteristic (ROC) curves with the corresponding C statistics (area under the curve (AUC) were developed to quantify the accuracy of the composite score of miRNAs together with that of each miRNA for predicting MPE vs. NMPE). A *p*-value of less than 0.05 was considered statistically significant. 

## 3. Results 

**Figure 1:**
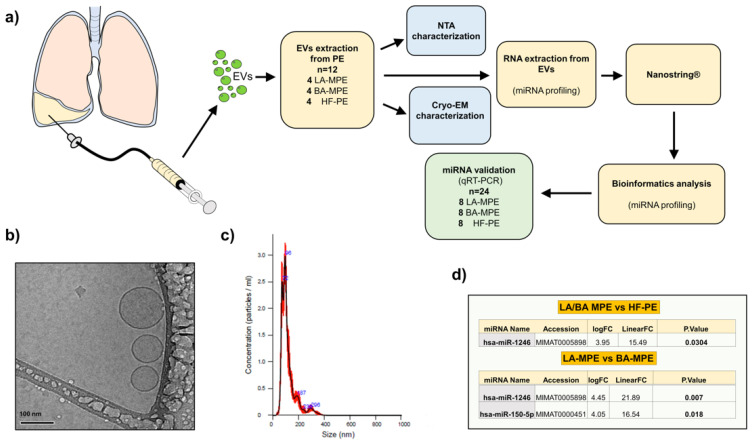
(**a**) Study schematic diagram with pictographic diagram of pleural fluid collection and EV extraction. (**b**) Cryo-EM images of extracellular vesicles isolated from pleural fluid and (**c**) nanoparticle tracking analysis (NTA). (**d**) Table of miRNA profiles: cancer (LA-MPE/BA-MPE) vs. HF-PE (top table) and breast vs. lung MPE (bottom table).

Isolated EVs from the effusions of 24 patients were examined for miRNA patterns of expression (16 metastatic adenocarcinoma (8 LA-MPE and 8 BA-MPE) and 8 controls with HF-PE) by a Nanostring nCounter^®^ miRNA expression panel. Patient characteristics are listed in Table 1. EVs were characterized using Nanoparticle Tracking Analysis and Cryogenic Electron Microscopy (Figure 1a–c). 

**Figure 2:**
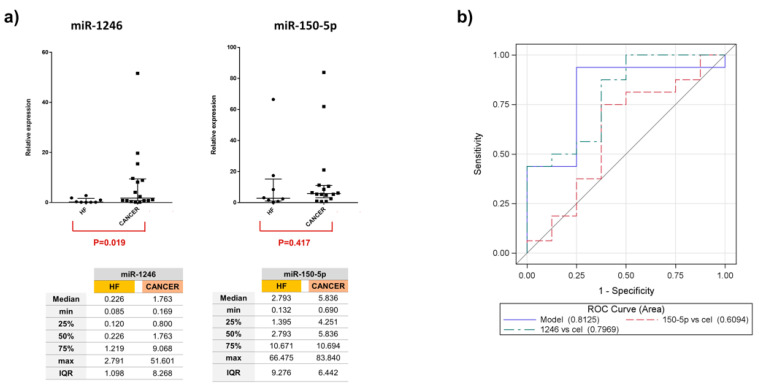
(**a**) Relative expression of miR-1246 and miR-150-5p in all 24 samples report median and interquartile range in the graphs. (**b**) ROC curves and AUC scores for miR-1246, miR-150-5p, and the composite of two miRNAs demonstrate a more superior AUC than each miRNA alone, in differentiating malignant vs. non-malignant effusion.

**Figure A1:**
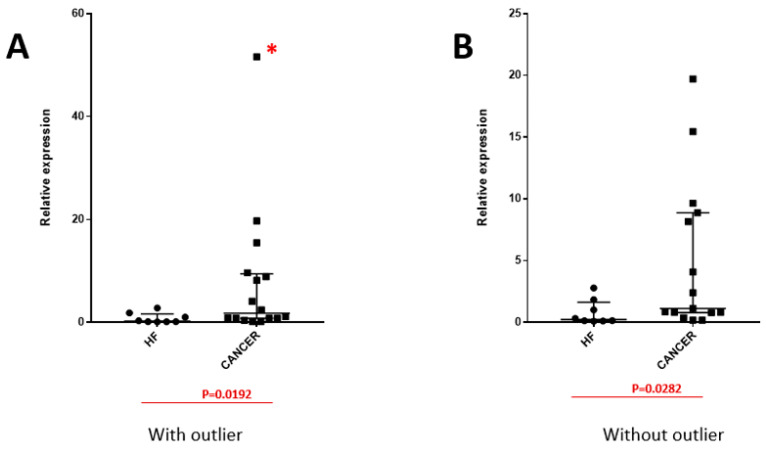
miR-1246 expression between cancer and non-cancer heart failure (HF)-induced pleural effusion with the presence of an extreme value of miR-1246, considered as a potential outlier (identified with a red *), as demonstrated in image (**A**) and after the exclusion of outlier value, as demonstrated in image (**B**).

EV microRNA differential expression analysis between all the groups (LA-MPE, BA-MPE, and HF-PE) was performed (Supplemental Table S1). We elected to validate only the upregulated miRs whose expression resulted in 20 counts in at least one condition. The differential expression analysis between cancer and control cases yielded one miRNA (miR-1246) that was significantly dysregulated among 12 patients (4 LA-MPE, 4 BA-MPE, and 4 HF-PE). Additionally, between breast and lung cancer, two miRNAs (miR-1246 and miR-150-5p) were significantly dysregulated (Figure 1d). To validate this differential expression, we used qRT-PCR to assess differentially expressed miRNAs in a total of 24 patients (the original 12 and a second, separate cohort of 12 patients from the same biorepository). The dysregulated miRNAs remained differentially expressed in cohorts of cancer (16) versus the HF-PE group (8). This difference was statistically significant for miR-1246 (*p* = 0.019) (Figure 2 and Appendix A, Figure A1). 

**Figure A2:**
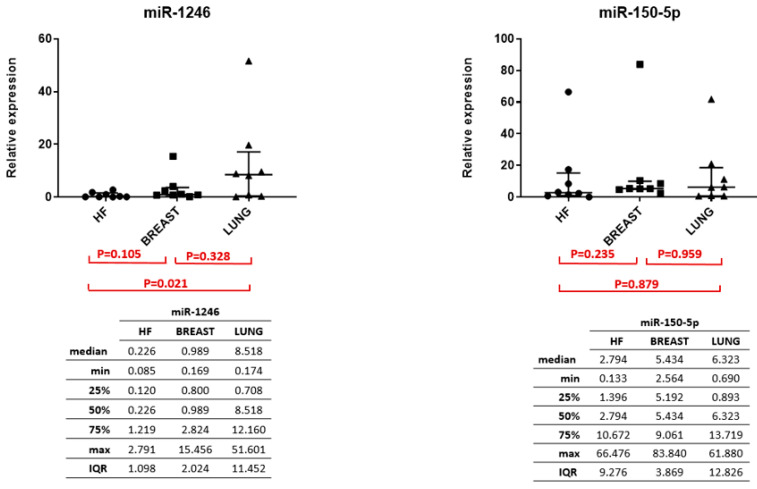
miR-1246 expression among LA-MPE, BA-MPE, and HF demonstrated in image (**A**), and miR-150-5p expression among LA-MPE, BA-MPE, and HF demonstrated in image (**B**) within the entire cohort.

Logistic models were developed to assess the statistical association of composite dysregulated miRNA, as compared to the association of each miRNA alone in MPE vs. NMPE. The combination of two miRNAs showed an AUC of 0.81 (95% CI: 0.61, 0.99) and was the best classifier. miRNA-1246 with an AUC of 0.79 (95% CI: 0.60, 0.99) presented a 4-fold increased expression (OR: 3.87, 95% CI: 0.43, 35), in MPE vs. NMPE (Figure 2b). miRNA 150-5p showed no significant variability in expression (OR: 0.95, 95% CI: 0.85, 1.04) in the MPE vs. control group. Comparing subgroups of LA-MPE, BA-MPE, and HF-PE in the entire population (three groups of eight), miR-1246 had a significantly higher expression in LA-MPE compared to the HF-PE groups (*p* = 0.02). The miRNAs were differentially expressed between LA-MPE and BA-MPE, although this difference did not reach statistical significance. Between-group differences for both miRNAs are shown in Appendix A, Figure A2. 

Finally, to further investigate the functional potential of these two EV-included miRNAs, we performed a miRNA-target consensus prediction analysis followed by a GO term-based functional enrichment analysis by employing the isoTar tool [12] (see Materials and Methods for more details). Globally, we found 863 and 211 predicted targets for the miR-150-5p and miR-1246, respectively. Furthermore, we also performed a GO analysis predicting 98 biological processes, 23 cellular components, and 38 molecular functions potentially impacted by the miR-150-5p and 41 biological processes, four cellular components, and 15 molecular functions by the miR-1246 (Supplemental Table S2).

## 4. Discussion

This exploratory study demonstrated that miR-1246 and miR-150-5p were differentially expressed in the pleural fluid of patients with LA-MPE and BA-MPE compared to HF-PE. The combination of miRNAs was the best classifier (AUC: 0.81) in differentiating malignant vs. HF-PE effusions. Additionally, miR-1246 had significantly higher expression in the LA-MPE vs. HF-EF group (*p* = 0.021). Both miRNAs were differentially expressed between LA-MPE and BA-MPE, although this difference did not reach statistical significance. Lung and breast adenocarcinoma are the two most common causes of MPE, and this study is the first to compare the miRNA expression between these two cancers in pleural fluid. 

In our population, miR-1246 expression was upregulated nearly four-fold in cancer versus the control group. These findings are consistent with the existing literature on miR-1246 expression in both lung and breast cancer tissue [18,19,20]. Upregulation of miR-1246 in both cancers demonstrated diagnostic utility compared to non-cancer control groups. Expression of miR-1246 showed a strong association with reduced event-free survival in early and metastatic breast cancer [20,21]. In non-small-cell lung cancer (NSCLC), patients with upregulation of miR-1246 had lower disease-free and overall survival and were more likely to develop advanced disease and lymph node metastasis [22]. Serum miR-1246 was an independent predictor of poor survival in multivariable analysis [21,22]. These data are consistent with our population, as MPE confirms metastatic stage IV disease.

Published evidence on the role of miR-150-5p in lung cancer has shown varying results but primarily demonstrates that dysregulation of miR-150 is associated with the proliferation of NSCLC [23,24,25]. In one study, miR-150-5p was significantly downregulated in cancer stem cells of NSCLC, and its expression level was negatively correlated with disease progression and poor survival in patients with NSCLC [24]. Similarly, Shunsuke et al. demonstrated downregulation of miR-150-5p and miR-150-3p in lung adenocarcinoma specimens [25]. Dysregulation of miR-150-5p in MPE was also shown by Roman-Canal et al. However, in their study, the investigators noted upregulation of miR-150-5p in patients with lung cancer compared to the NMPE group [8]. 

Our study confirms that pleural fluid can be a source of liquid biopsy for the investigation of EV-miRNA in lung and breast cancer. Importantly, compared to prior similar studies that have included heterogeneous groups, this homogeneous dataset represents three specific diseases (BA-MPE, LA-MPE, and HF-PE), which were defined based on clinical criteria, and which can be confirmed in similar populations in future prospective studies. Blood and sputum represent the two primary sources for liquid biopsy in the oncology literature. Pleural fluid, as a new source, adds a significant advantage due to direct access to malignant cells and their microenvironment. In the setting of lung and breast cancer, transcutaneous fine-needle aspiration (FNA) and bronchoscopic-guided lavage, brush, and FNA are possible but are more invasive and not repeatedly indicated. Pleural fluid drainage, however, is indicated to provide symptomatic relief and assist with diagnosis and staging. Ongoing access to the fluid allows for the opportunity to study tumor cell progression and evolution. 

The most important limitation of this study is the sample size. This was an exploratory pilot study and its results require confirmation in larger cohorts. Despite its small size, this study included three homogeneous groups and provides preliminary data for effect size and power calculation in larger follow-up investigations. Due to the small number of groups, within-group comparisons and adjustments for confounding variables may not be reliable.

In summary, we discovered distinct miRNA signatures that appear to reliably identify a malignant pleural process and may be deregulated according to tissue type. These findings require prospective validation in larger datasets with identical clinical diagnostic criteria. The downstream function of these biomarkers and their role in tumor escape, migration, invasion, and metastasis are unknown and merit further investigation.

## Figures and Tables

**Table 1:** Patients’ characteristics.

Variables		Malignant (16)	Non-Malignant (8)	
Age	Mean (SD)	61.8 (12.3)	76.4 (7.3)	
Sex				
Males		3 (19%)	4 (50%)	
Females		13 (81%)	4 (50%)	
Race				
White		12 (75%)	4 (50%)	
Black		4 (25%)	4 (50%)	
Smoking Status				
Former		6 (37%)	4 (50%)	
Never		7 (44%)	4 (50%)	
Current		3 (19%)	0 (0%)	
ECOG				
0		1 (6%)	0 (0%)	
1		6 (37%)	2 (25%)	
2		5 (31%)	3 (37%)	
3		3 (19%)	3 (37%)	
4		1 (6%)	0 (0%)	
Etiology				
Lung Adeno.		8 (50%)	CHF	8 (100%)
Breast Adeno		8 (50%)		

SD: standard deviation, ECOG: Eastern Cooperative Oncology Group, Adeno: adenocarcinoma, CHF: congestive heart failure.

## Data Availability

Raw data are available immediately upon request.

## Supplementary Materials

The following supporting information can be downloaded at: https://www.mdpi.com/article/10.3390/genes13112159/s1, Table S1: The list of the expressed miRNAs in all the comparisons. Table S2: Analysis of miR-150-5p and miR-1246 the predicted targets.

## Abbreviations

MPE	Malignant pleural effusion
NMPE	Non-malignant pleural effusion
miRNAs	microRNAs
EVs	Extracellular vesicles
HF-PE	Heart-failure-induced effusions
LA-MPE	MPE due to lung adenocarcinoma
BA-MPE	MPE due to breast adenocarcinoma
SD	Standard deviation
IQR	Interquartile range
ROC	Receiver operating characteristic
AUC	Area under the curve
qRT-PCR	Quantitative Reverse Transcription Polymerase Chain Reaction
CI	Confidence interval
FNA	Fine-needle aspiration
NSCLC	Non-small-cell lung cancer

## Appendix A. Aspiration Procedures

All thoracenteses were performed under the supervision of an attending physician from the interventional pulmonology team and in a pleural disease negative-pressure room. Portable ultrasound was used for examination of the pleural space and puncture-site identification. Operator/operators were geared with sterile gown gloves and wore surgical cap, mask, and face shield. The identified puncture site, including up to at least 10 cm in diameter radius of the surrounding area, was prepped twice, each time using ChloraPrep (2% chlorhexidine gluconate in 70% isopropyl alcohol). Following sterile prep, a sterile drape was placed on the torso of the patient, only exposing the puncture site and small portion of the surrounding sterile prepped skin. With the use of 1% lidocaine, local analgesia of the puncture site and deep tissue was achieved. Following this, a soft thoracentesis pleural catheter was inserted and the needle withdrawn using the standard technique (Arrow-Clarke Pleura-Seal Sterile Thoracentesis Kit). Pleural fluid was removed by manual aspiration.
